# Targeted plasma metabolomics combined with machine learning for the diagnosis of severe acute respiratory syndrome virus type 2

**DOI:** 10.3389/fmicb.2022.1059289

**Published:** 2023-03-29

**Authors:** Anthony T. Le, Manhong Wu, Afraz Khan, Nicholas Phillips, Pranav Rajpurkar, Megan Garland, Kayla Magid, Mamdouh Sibai, ChunHong Huang, Malaya K. Sahoo, Raffick Bowen, Tina M. Cowan, Benjamin A. Pinsky, Catherine A. Hogan

**Affiliations:** ^1^Department of Pathology, Stanford University School of Medicine, Stanford, CA, United States; ^2^Department of Anesthesiology, Stanford University School of Medicine, Stanford, CA, United States; ^3^British Columbia Center for Disease Control Public Health Laboratory, Vancouver, BC, Canada; ^4^Stanford Computer Science Department, Stanford University, Stanford, CA, United States; ^5^Clinical Chemistry and Immunology Laboratory, Stanford Health Care, Palo Alto, CA, United States; ^6^Stanford Biochemical Genetics Laboratory, Stanford Health Care, Palo Alto, CA, United States; ^7^Stanford Clinical Virology Laboratory, Stanford Health Care, Palo Alto, CA, United States; ^8^Division of Infectious Diseases and Geographic Medicine, Department of Medicine, Stanford University School of Medicine, Stanford, CA, United States; ^9^Department of Pathology and Laboratory Medicine, University of British Columbia, Vancouver, BC, Canada

**Keywords:** plasma, metabolomics, amino acids, SARS-CoV-2, COVID-19

## Abstract

**Introduction:**

The routine clinical diagnosis of severe acute respiratory syndrome coronavirus 2 (SARS-CoV-2) is largely restricted to real-time reverse transcription quantitative PCR (RT-qPCR), and tests that detect SARS-CoV-2 nucleocapsid antigen. Given the diagnostic delay and suboptimal sensitivity associated with these respective methods, alternative diagnostic strategies are needed for acute infection.

**Methods:**

We studied the use of a clinically validated liquid chromatography triple quadrupole method (LC/MS–MS) for detection of amino acids from plasma specimens. We applied machine learning models to distinguish between SARS-CoV-2-positive and negative samples and analyzed amino acid feature importance.

**Results:**

A total of 200 samples were tested, including 70 from individuals with COVID-19, and 130 from negative controls. The top performing model overall allowed discrimination between SARS-CoV-2-positive and negative control samples with an area under the receiver operating characteristic curve (AUC) of 0.96 (95%CI 0.91, 1.00), overall sensitivity of 0.99 (95%CI 0.92, 1.00), and specificity of 0.92 (95%CI 0.85, 0.95).

**Discussion:**

This approach holds potential as an alternative to existing methods for the rapid and accurate diagnosis of acute SARS-CoV-2 infection.

## Introduction

Severe acute respiratory syndrome virus type 2 (SARS-CoV-2) is the causative agent of coronavirus disease 2019 (COVID-19) and continues to spread globally despite the availability of effective vaccines (European Centre for Disease Prevention and Control, 2021). Therefore, early diagnosis is crucial to identify infected individuals to provide therapy, if indicated, and implement appropriate infection control measures to prevent and limit spread. Real-time reverse transcription quantitative PCR (RT-qPCR) represents the operational gold standard for diagnosis of acute SARS-CoV-2 infection; however, such testing may suffer from long turnaround times, particularly during surges, and requires reagents and consumables that have been regularly compromised since the onset of the pandemic (Kucirka et al., 2020; Woloshin et al., 2020). Similarly, SARS-CoV-2 antigen testing can provide a rapid means to results *via* point-of-care testing, but typically requires several days from the onset of symptoms for detection, and sensitivity varies substantially based on the device used. Though SARS-CoV-2 preferentially infects upper respiratory epithelial cells, COVID-19 is a systemic illness that may induce specific amino acid alterations in the host (Sungnak et al., 2020; Mulay et al., 2021). These metabolomic changes may then be harnessed as a diagnostic approach that detects host response to infection rather than the virus itself. This approach of analyzing amino acids in plasma or serum to diagnose of COVID-19 has been previously pursued, but with heterogeneous methodologies, largely not validated clinically (Fraser et al., 2020; Shen et al., 2020; Thomas et al., 2020). Furthermore, machine learning has emerged as a powerful tool for classification analysis in metabolomics data analysis (Mendez et al., 2019; Dias-Audibert et al., 2020; Shen et al., 2020; Delafiori et al., 2021). In this study, we adapted a clinically validated amino acid quantitation method to differentiate SARS-CoV-2-positive from negative samples from plasma and to identify the top differentiating amino acid biomarkers associated with classification performance by statistical and machine learning models.

## Materials and methods

### Ethics

This study was approved by the Stanford Institutional Review board (IRB protocol #57519).

### Study population and sample collection

We identified individuals with RT-qPCR-confirmed SARS-CoV-2 infection from a respiratory sample (nasopharyngeal, nasal, or oropharyngeal). Participants were selected from two academic tertiary care hospitals [Stanford Health Care (SHC) and Lucille Packard Children’s Hospital (LPCH)] and affiliated clinics and outpatient centers in the Bay Area, from March 2020 to November 2020. SARS-CoV-2 testing was performed as previously described, using an in-house emergency use authorization (EUA) real-time reverse transcription PCR (RT-qPCR), or one of two commercial SARS-CoV-2 assays, the Panther Fusion or TMA (Hologic, Malborough, MA, United States; Food and Drug Administration, 2020). Residual plasma specimens were obtained from individuals with confirmed SARS-CoV-2 infection and used for plasma metabolomics testing. Due to the requirement for a blood draw, sample selection was largely restricted to hospitalized individuals. In addition, most nasopharyngeal testing was performed on symptomatic individuals during the study time period. Only plasma samples collected within 7 days of the initial SARS-CoV-2 infection were included to include acute COVID-19, and there was no additional selection based on cycle threshold (Ct) value or clinical severity. In addition, we identified individuals to serve as negative controls from the following groups: pooled donor blood negative for SARS-CoV-2, hospitalized individuals and outpatients with residual plasma from EBV or CMV viral load testing, hospitalized individuals with elevated C-reactive protein (CRP), and/or procalcitonin (PCT) and without SARS-CoV-2 infection, and symptomatic individuals with a confirmed respiratory viral infection other than SARS-CoV-2. For the latter group, respiratory viral testing was performed on the ePlex Respiratory Pathogen (RP) panel (GenMark Diagnostics, Carlsbad, CA, United States) at the Stanford Clinical Virology Laboratory. C-reactive protein (CRP) is a protein synthesized by the liver that can acutely rise in response to inflammation and is readily tested through routine testing in clinical laboratories. Procalcitonin (PCT) is the peptide precursor of calcitonin, which is synthesized by the thyroid gland, and positively correlates with bacterial infection and sepsis. Both biomarkers, CRP and PCT, were examined to help understand the specificity of the generated plasma amino acid signature. Given that plasma is not a routinely collected specimen for the diagnosis of COVID-19, we enrolled eligible individuals without matching for age and sex between the positive and negative groups. Plasma procalcitonin (PCT) and C-reactive protein (CRP) concentrations were measured on a Roche Cobas e801 and c702 modules, respectively (Roche Diagnostics, Indianapolis, IN, United States).

### Underivatized amino acid analysis by LC/MS–MS

As previously described, amino acids were quantified by LC/MS-MS using a clinically validated method (Le et al., 2014). In brief, a volume of 20 μl of plasma was mixed with an equal volume of 6% sulfosalicyclic acid and then centrifuged at 4°C for 15 min at 17,000 × *g*. Twenty μl of the supernatant was mixed with 1.4 ml of an internal standard mixture in a 96-well plate, which was prepared as previously described (Mak et al., 2019). Testing was performed on an Agilent 6460 Tandem Mass Spectrometer with electrospray ionization (Agilent Technologies, Santa Clara, CA, United States). Chromatographic separation was performed using a series of two columns: column 1, a porous graphitic carbon (PGC) column (Thermo Fisher Scientific, 3 μm Hypercarb, 4.6 mm ID × 50 mm), and column 2, an XBridge BEH C18, (Waters Corp, 2.5 μm, 2.1 mm ID × 100 mm). An injection volume of 5 μl was used; with a runtime of 13.5 min. Compounds were analyzed in positive-ion mode and detected by scheduled selective reaction monitoring (SRM). Data were acquired using MassHunter Workstation Acquisition version B.08.02 (Agilent), analyzed by MassHunter Quant software version B.07.00 (Agilent), and exported to Microsoft Excel version 15.0.5501.1000. Quantitative analysis was performed by relating chromatographic peak areas to those derived from externally run calibration standards as described above and normalized using isotopic-labelled internal standards (Cambridge Isotope Laboratories, Metabolomics Amino Acid Mix Standard MSK-A2-1.2). Calibration curves were plotted using a weighted regression 1/x (Le et al., 2014). This method was developed based on the standards of the Clinical Laboratory Improvement Amendments (CLIA), and is CLIA-certified.

### Statistical analysis

Descriptive analysis was performed by Chi-squared test or Fisher’s exact test for variables with less than five data points per cell, and Mann–Whitney *U* test for continuous variables, using Stata v15.1 (Stata Corp, College Station, TX, United States). A multivariable analysis was used to investigate the significance of the *a priori* determined potential confounder’s age and sex in the analysis, as previously described using R version 4.0.2 (Hogan et al., 2021). The significance of each predictor was determined using the value of *p* from this regression.

### Machine learning analysis

Machine learning analysis was performed as previously described (Hogan et al., 2021). The full dataset was randomly divided into a training set (70% of samples) used to develop machine learning models, and a holdout test set (30% of samples) used to evaluate the predictive performance of the machine learning models. The SHapley Additive exPlanations (SHAP) method was used to quantify the impact of each feature on the models. Analyses were performed in Python version 3.7.10, using the LightGBM v3.1.0 implementation for gradient boosted decision trees, scikit-learn v0.23.2 for random forest, stratified *k*-fold cross-validation and grid search, and SHapley Additive exPlanations (SHAP) v0.36.0 for computing feature importance, using the code shared online for reproducibility.

## Results

### Cohort description

A total of 200 samples were included in the study, including 70 samples from individuals with confirmed SARS-CoV-2 infection, and 130 samples from negative controls (Supplementary Figure 1). Of these, 23 negative control samples represented pooled samples from blood donors for which individual-level data were not available. The baseline demographic and clinical characteristics of the patient cohort are described in Table 1. Briefly, the overall median age was 53 years (36–67), and almost half (46.3%) of participants were female.

**Table 1:** Demographic, clinical, and laboratory characteristics of the individuals included in the study.

		Overall (*n* = 177)	Negative for COVID-19 (*n* = 107)	Positive for COVID-19 (*n* = 70)	Value of p^*^
Median age (IQR)		53 (36–67)	56 (32.5–66.5)	49.5 (39.3–66.8)	0.8
Age [No. (%)]	≥2–17 yo	19 (10.7)	16 (15.0)	3 (4.3)	0.03
	≥18 yo	158 (89.3)	91 (85.0)	67 (95.7)	
Sex [No. (%)]	Male	95 (53.7)	63 (58.9)	38 (54.3)	0.5
	Female	82 (46.3)	44 (41.1)	32 (45.7)	
Elevated C-reactive protein [No. (%)]	Yes	71 (40.1)	43 (40.2)	28 (40.0)	1
	No	9 (5.1)	1 (0.9)	8 (11.4)	
	Unknown	97 (54.8)	63 (58.9)	34 (48.6)	
Elevated procalcitonin [No. (%)]	Yes	27 (15.3)	33 (30.8)	4 (5.7)	0.0001
	No	113 (63.8)	4 (3.7)	23 (32.9)	
	Unknown	37 (20.9)	70 (65.4)	43 (61.4)	

* Chi-squared test, Fisher’s exact test, or Mann Whitney U test.

Data exclude 23 samples from adults donating blood for whom individual-level data were not available.

### Targeted plasma amino acid data classification and feature ranking analysis

**Figure 1:**
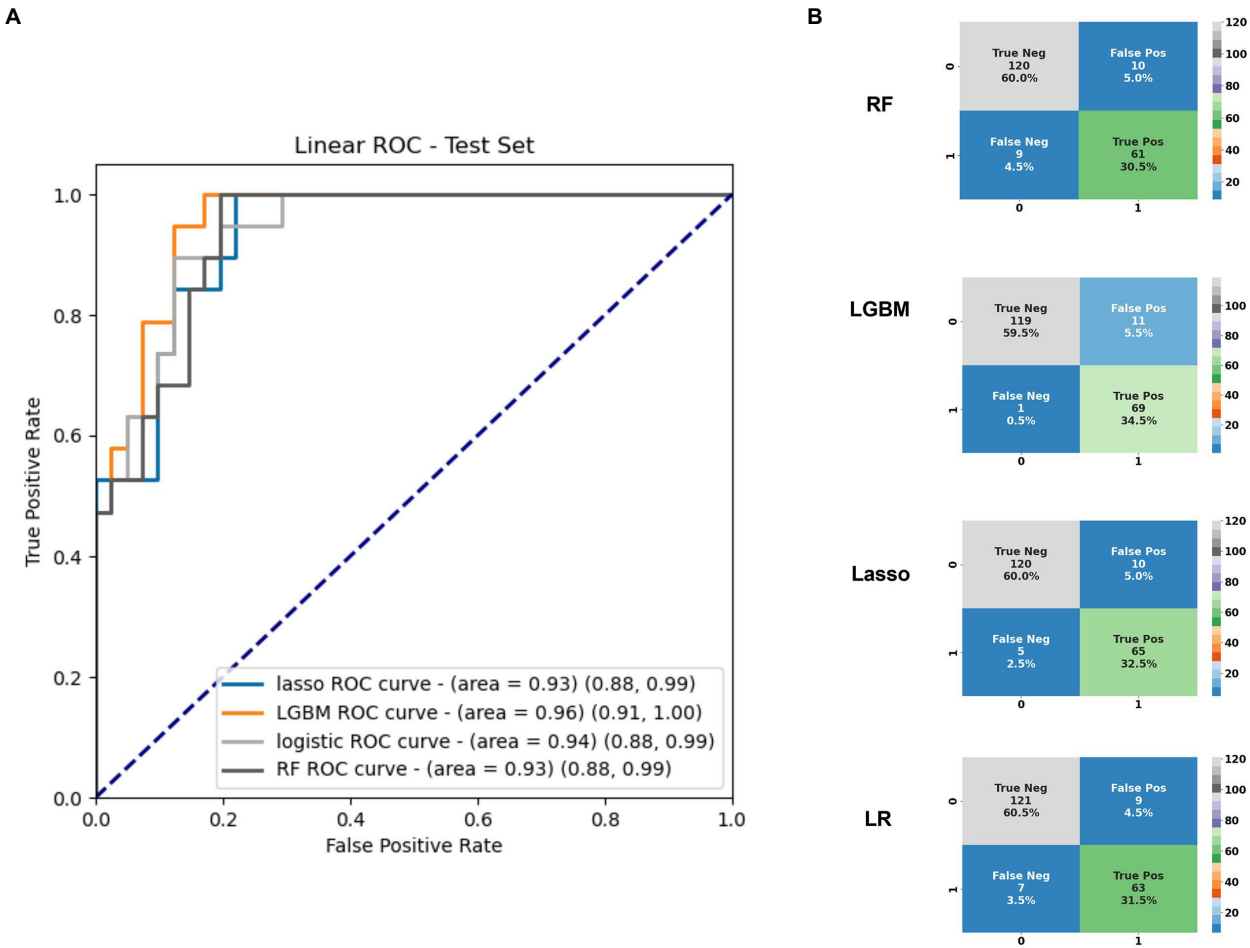
**(A)** Area under the receiver operating characteristic curve for the top 20 amino acids based on the test set identified in plasma differentiating infected from uninfected individuals, and **(B)** Confusion matrices based on the full cross-validation for each of the four models used. AUC, area under the receiver operating characteristic curve; LGBM, Light Gradient Boosted Model; LR, logistic regression; Ped, pediatric; RF, random forests; and ROC, receiver operating characteristic curve.

**Figure 2:**
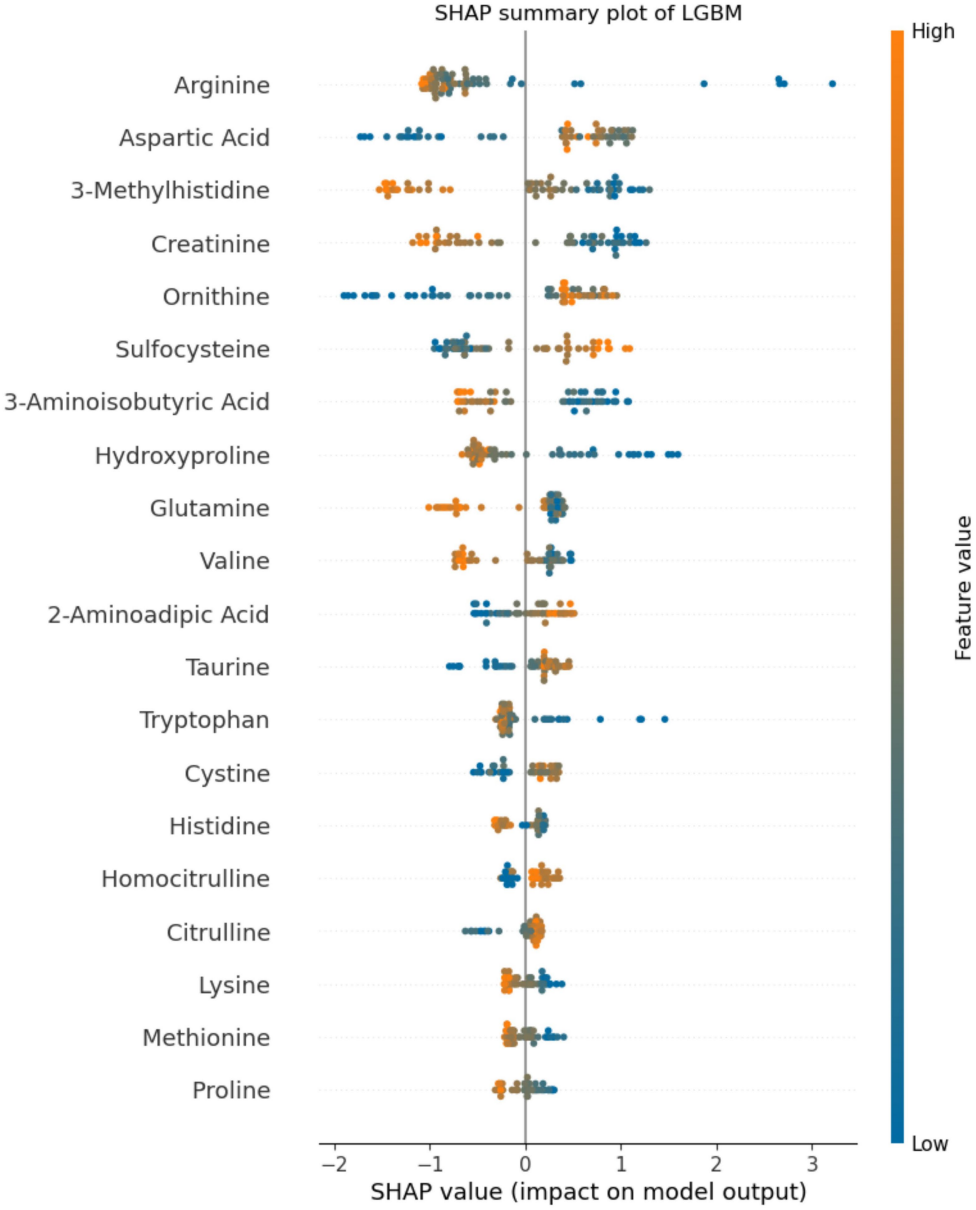
Feature importance analysis by SHapley Additive exPlanation (SHAP) values. The top 20 amino acids by percentage importance using the SHAP method are presented by amino acid. The colors indicate the association between feature value and positive SARS-CoV-2 classification, with features pushing the risk of SARS-CoV-2 higher in blue, and features pushing the risk of SARS-CoV-2 lower in orange. The axis scale represents the predicted SHAP output value scale. Positive SHAP values indicate positive impact on model prediction (leading the model to predict SARS-CoV-2-positive), whereas negative SHAP values indicate negative impact on model prediction (leading the model to predict SARS-CoV-2-negative).

Application of statistical (Lasso, logistic regression) and machine learning (Random Forests, LGBM) models to the plasma amino acids tested features achieved a maximal area under the receiver operating characteristic curve (AUC) of 0.96 (95%CI 0.91, 1.00) on the test set with the LGBM model (Table 2), which was also the best performing model overall. At an operating cut-off optimized for sensitivity, this model achieved an overall sensitivity of 0.99 (95%CI 0.92, 1.00) and specificity of 0.92 (95%CI 0.85, 0.95; Figure 1). The separate multivariable model adjusting for age and sex demonstrated that only model outcome was significantly associated with SARS-CoV-2 infection status (Supplementary Table 1). Feature importance ranking by SHAP analysis on the LGBM model revealed that arginine, aspartic acid, and 3-methylhistidine were the top amino acid biomarkers associated with model classification performance (Figure 2). Furthermore, although not as strongly associated with classification, tryptophan was decreased (34.8 in infected vs. 45.8 in negative; *p* < 0.0001) in individuals with acute COVID-19. Median concentration levels and distribution of values revealed that the largest relative differences were observed in arginine (32.7 in infected vs. 87.2 in negative samples; *p* < 0.0001) and sulfocysteine (5.48 in infected vs. 3.37 in negative; *p* < 0.0001; Supplementary Table 2 and Supplementary Figure 2). Stratification of these results by CRP status revealed that arginine concentration was highest in the high CRP/COVID-negative subgroup, and that the levels across other subgroups were similar (Supplementary Figure 3). Similarly, the lowest sulfocysteine concentration was observed in the high CRP/COVID-negative subgroup, whereas other subgroups were similar.

**Table 2:** Summary of area under the curve, sensitivity, specificity data for the two machine learning, and two statistical models used for the study.

	LGBM	Random Forests	Lasso	Logistic regression
AUC(95% CI)	0.96 (0.91, 1.00)	0.93 (0.88, 0.99)	0.93 (0.88, 0.99)	0.94 (0.88, 0.99)
Sensitivity(95% CI)	0.99 (0.92, 1.00)	0.87 (0.77, 0.93)	0.93 (0.84, 0.97)	0.90 (0.81, 0.95)
Specificity(95% CI)	0.92 (0.85, 0.95)	0.92 (0.86, 0.96)	0.92 (0.86, 0.96)	0.93 (0.87, 0.96)

AUC, area under the receiver operating characteristic curve; CI, confidence interval; and LGBM, light gradient boosted model

## Discussion

In this study, we showed that the described targeted amino acid method combined with machine learning could differentiate between SARS-CoV-2-positive and SARS-CoV-2-negative samples with high test performance, including AUC of 0.96 and sensitivity of 0.99. Of the 54 amino acids tested, 3-methylhistidine, arginine, and glutamine were the top differentiating amino acids. Several studies have investigated plasma metabolomics for the diagnosis of SARS-CoV-2 infection. However, testing methodologies have varied substantially, spanning several untargeted and targeted mass spectrometry approaches and with heterogeneous patient populations (Blasco et al., 2020; Fraser et al., 2020; Meoni et al., 2021; Zhang et al., 2021), generating broad understanding but limiting result comparability and generalizability. An early proteomic and metabolomic study in the COVID-19 pandemic documented suppression of over 100 amino acids and their derivatives in the serum of individuals diagnosed with COVID-19, particularly involving arginine metabolism (Shen et al., 2020). The current study distinguishes itself based on using a robust, clinically validated method. Using this approach, we showed both elevated (including aspartic acid and sulfocysteine) and decreased (including arginine, 3-methylhistidine, creatinine, and tryptophan) amino acid levels in the plasma of individuals with acute COVID-19. These divergent findings may have occurred due to different sample processing methodologies (ethanol and drying followed by methanol extraction vs. sulfosalicylic acid precipitation), or testing methods (untargeted UPLC-MS/MS vs. targeted LC/MS–MS). Subsequent work has demonstrated variable amino acid findings. However, an interesting finding shared across several studies has been a decrease in tryptophan and an increase in kynurenine in the serum and plasma of SARS-CoV-2-infected individuals, which may be more pronounced in severely ill individuals (Fraser et al., 2020; Shen et al., 2020; Thomas et al., 2020; Lawler et al., 2021; Lionetto et al., 2021; Mangge et al., 2021; Cihan et al., 2022). Importantly, the current study corroborated this decrease in tryptophan, adding strength to the signal found in the literature. This study did not assess kynurenine, given that this amino acid is not quantified with standards in the present method.

At a cut-off selected to optimize sensitivity, this study documented a sensitivity of 0.99 for a specificity of 0.92. This test performance, combined with the employed method’s simple sample processing, rapid turnaround time, and potential for high throughput, supports the potential of this approach as a screening test. Indeed, one potential avenue of testing for individuals undergoing assessment in a hospital setting and requiring a blood draw would be to screen plasma for SARS-CoV-2 using this targeted plasma approach as a rapid rule-out test. Suspect samples could undergo further SARS-CoV-2 testing by respiratory testing, and negative samples could be presumptively ruled-out unless there is high suspicion for clinical or epidemiological reasons.

The main strength of this study is the use of a clinically validated LC/MS–MS method for reliable amino acid quantitation. Data generated from similarly validated quantitative amino acid methods run in other laboratories would also be useful to advance the field. Furthermore, the study benefited from a large sample size and incorporated assessment of CRP level in a subset of individuals. Stratification of the metabolomics results by CRP contributed to assessment of the specificity of the biomarker signature in assessing viral-specific vs. general inflammatory response. However, there are limitations. First, only individuals with confirmed SARS-CoV-2 from a respiratory source and residual plasma samples were included; as such, we could not adjust for time since infection and onset of symptoms, or comprehensively study other respiratory viruses, in the same manner as a prospective study. Second, due to the observational design of the study, we could not assess the effect of longitudinal sampling, COVID-19 disease severity, vaccination status, full CRP and PCT characterization, additional variants of concern, and treatment responses, all of which require additional study. Third, the direct clinical application of plasma-based testing may be more limited due to its more invasive nature than respiratory sampling and the requirement for a healthcare provider-based procedure. However, this specimen type is attractive given that the metabolites are expected to be present in much higher concentrations in the bloodstream than in respiratory sites, and due to the greater standardization of sample collection, which may enhance reproducibility of results. Finally, the current results do not support replacement of standard COVID-19 diagnostic approaches such as RT-qPCR. Rather, these preliminary data support the potential complementary value of this method, especially as a tool for pathway analysis, compound identification and for clinical prognostication, which will require further investigation.

In summary, we demonstrated the high accuracy of a clinically validated LC/MS–MS analysis combined with machine learning for amino acid profiling in SARS-CoV-2-positive and negative plasma specimens. This approach holds potential for screening suspect cases, given its high sensitivity. Further work to validate this amino acid signature in other patient populations and in respiratory specimens, and using methods validated with a similar rigor in additional laboratories, will further complement these findings.

## Data availability statement

The data presented in the study are deposited in the Metabolights repository, the accession number is MTBLS6739.

## Ethics statement

The studies involving human participants were reviewed and approved by Stanford Institutional Review board (IRB protocol #57519). Written informed consent from the participants’ legal guardian/next of kin was not required to participate in this study in accordance with the national legislation and the institutional requirements.

## Author contributions

CHo, AL, AK, TC, and BP: conceptualization. AK, NP, CHo, AL, and MW: data curation, validation, and writing—original draft. AL and TC: methodology. AK, PR, and NP: software. AK, NP, and CHo: formal analysis. CHo, AL, CHu, MSa, MG, KM, and MSi: investigation. BP, RB, and TC: resources. CHo, PR, AL, BP, TC, MW, and RB: writing—review and editing. BP and TC: supervision and funding acquisition. All authors contributed to the article and approved the submitted version.

## Funding

This work was funded by the Stanford Department of Pathology, and by Genome BC, Michael Smith Health Research BC, and British Columbia Centre for Disease Control Foundation.

## Conflict of interest

A provisional patent covering the machine learning analysis for metabolomics diagnostics has been filed (CHo, PR, AL, TC, BP).

The remianing authors declare that the research was conducted in the absence of any commercial or financial relationships that could be construed as a potential conflict of interest.

## Publisher’s note

All claims expressed in this article are solely those of the authors and do not necessarily represent those of their affiliated organizations, or those of the publisher, the editors and the reviewers. Any product that may be evaluated in this article, or claim that may be made by its manufacturer, is not guaranteed or endorsed by the publisher.

## Supplementary material

SUPPLEMENTARY TABLE 1 Multivariable linear regression model for SARS-CoV-2 status prediction adjusted for age, sex, and machine learning output. Model output was observed to be the most significant feature associated with infection status prediction.

SUPPLEMENTARY TABLE 2 Median concentrations of the top 20 amino acids in COVID-19 positive and negative samples.

SUPPLEMENTARY FIGURE 1 Flowchart of the specimen selection for assessment of the plasma targeted amino acid method. SARS-CoV-2: severe acute respiratory syndrome type.

SUPPLEMENTARY FIGURE 2 Amino acid concentration by LC/MS–MS standard curve analysis in SARS-CoV-2-positive vs. negative specimens for the top 20 differentiating amino acids.

SUPPLEMENTARY FIGURE 3 Amino acid concentration by LC/MS–MS standard curve analysis in SARS-CoV-2-positive vs. negative specimens for the top 20 differentiating amino acids, stratified by C-reactive protein status. The x-axis categories are listed in the following left to right order: Negative/high CRP, Negative/normal CRP, COVID/high CRP, COVID/normal CRP. CRP: C-reactive protein.
