# Supplementary material for: Targeted plasma metabolomics combined with machine learning for the diagnosis of severe acute respiratory syndrome virus type 2
Source: Front Microbiol. 2023 Mar 29;13:1059289. doi: 10.3389/fmicb.2022.1059289 (PMC10092816; doi:10.3389/fmicb.2022.1059289)
Supplement: SUPPLEMENTARY TABLE 1 — Multivariable linear regression model for SARS-CoV-2 status prediction adjusted for age, sex, and machine learning output. Model output was observed to be the most significant feature associated with infection status prediction. [file Data_Sheet_1.pdf]

Supplementary Material for:

Targeted plasma metabolomics combined with machine learning for the diagnosis of acute SARS-CoV-2

Le AT[1], Wu M[2], Khan A[3], Philips N[4], Rajpurkar PS[4,†], Garland M[1], Magid K[1], Sibai M[1], Huang C[1], Sahoo MK[1], Mak J[5], Bowen R[1,6], Cowan T[1,5], Pinsky BA[1,7,8], Hogan CA[1,3,7,9*]

[1]Department of Pathology, Stanford University School of Medicine, Stanford, CA, USA

[2]Department of Anesthesiology, Stanford University School of Medicine, Stanford, CA, USA

[3]British Columbia Centre for Disease Control Public Health Laboratory, Vancouver, BC, Canada

[4]Stanford Computer Science Department, Stanford University, Stanford, CA

[5]Stanford Biochemical Genetics Laboratory, Stanford Health Care, Palo Alto, CA, USA

[6]Clinical Chemistry and Immunology Laboratory, Stanford Health Care, Palo Alto, CA, USA

[7]Clinical Virology Laboratory, Stanford Health Care, Palo Alto, CA, USA

[8]Division of Infectious Diseases and Geographic Medicine, Department of Medicine, Stanford University School of Medicine, Stanford, CA, USA

[9]University of British Columbia, Vancouver, BC, Canada

[*]Address correspondence to Catherine A. Hogan, catherine.hogan@bccdc.ca

[†]Current affiliation: Department of Biomedical Informatics, Harvard Medical School, Boston, MA, USA

Table of contents

Supplemental Table 1. 3
Supplemental Table 2. 4
Supplemental Figure 1. 5
Supplemental Figure 2. 7
Supplemental Figure 3. 9

Supplemental Table 1. Multivariable linear regression model for SARS-CoV-2 status prediction adjusted for age, sex, and machine learning output. Model output was observed to be the most significant feature associated with infection status prediction.

	**Coefficient**	**Standard error**	**P-value**	**95% CI**
Age	0.0010	0.008	0.9	-0.0015, 0.017
Sex	-0.7566	0.340	0.03	-1.424, -0.089
LGMB model output	-1.45	0.354	<0.001	-2.148, -0.761

CI: confidence interval; LGBM: Light gradient boosted model

Supplemental Table 2. Median concentrations of the top 20 amino acids in COVID-19 positive and negative samples

Compound**	Median concentration (IQR)		Positive: negative ratio	p-value*
	Positive	Negative		
Arginine	33	87	0.38	1.6e-13
Aspartic acid	14	11	1.27	0.2
3-Methylhistidine	3	7	0.43	2.0e-11
Creatinine	58	91	0.64	4.7e-09
Ornithine	107	110	0.97	0.6
Sulfocysteine	5	3	1.67	1.2e-09
3-Aminoisobutyric acid	3	4	0.75	5.4e-07
Hydroxyproline	8	15	0.53	2.4e-08
Glutamine	391	439	0.89	0.03
Valine	240	345	0.70	2.2e-09
2-Aminoadipic acid	1	1	1	0.8
Taurine	131	93	1.41	0.009
Tryptophan	35	46	0.76	4.1e-06
Cystine	24	21	1.14	0.3
Histidine	66	86	0.77	6.4e-08
Homocitrulline	0	0	0	NA
Citrulline	21	26	0.81	0.01
Lysine	165	244	0.68	2.9e-08
Methionine	20	33	0.61	4.8e-08
Proline	151	250	0.60	1.2e-07

*Mann-Whitney *U* test
Concentration of compounds is listed in μM with the exception of pyroglutamic acid, which is listed in peak area abundance.

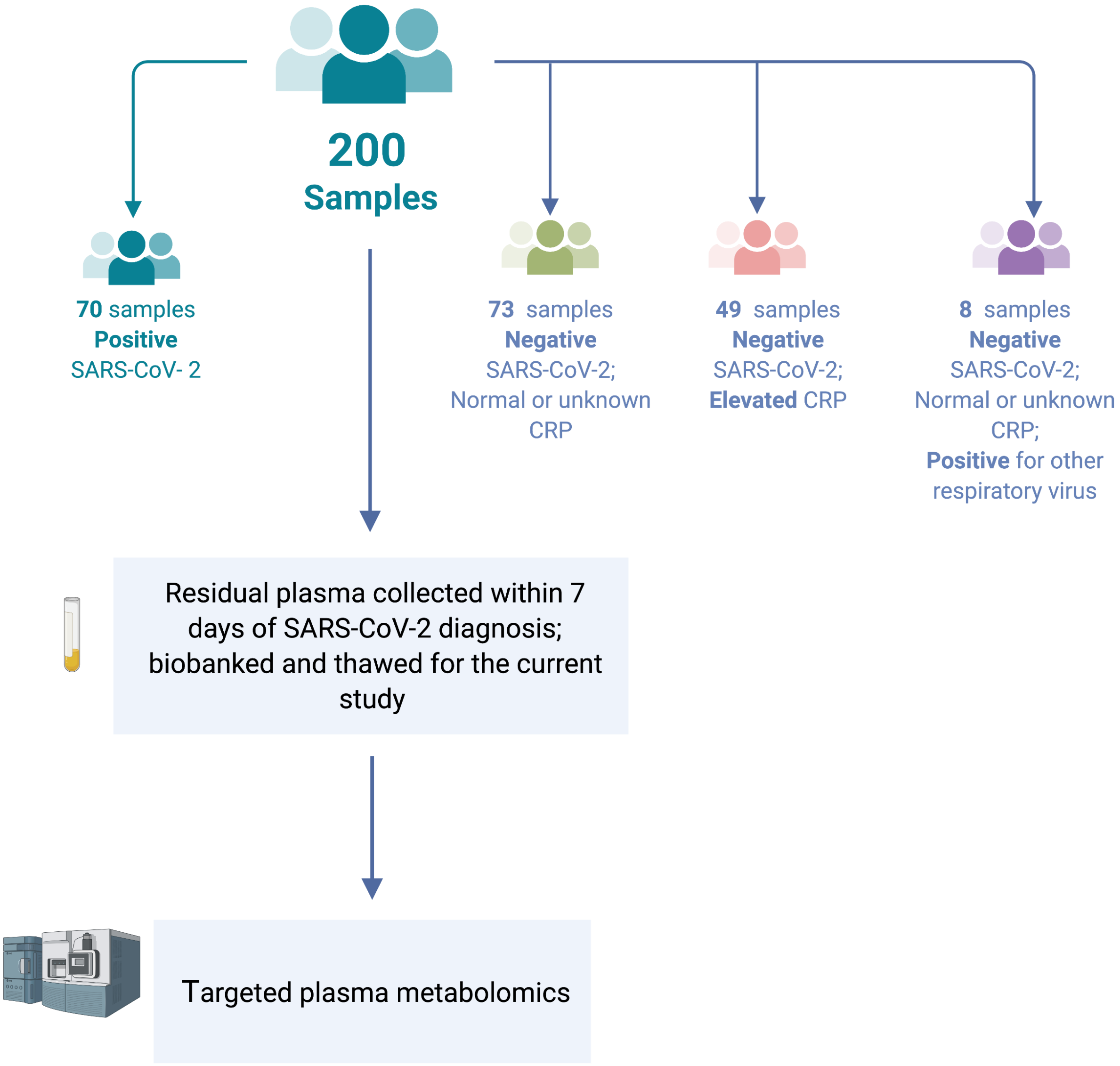

Supplemental Figure 1. Flowchart of the specimen selection for assessment of the plasma targeted amino acid method.

SARS-CoV-2: severe acute respiratory syndrome type

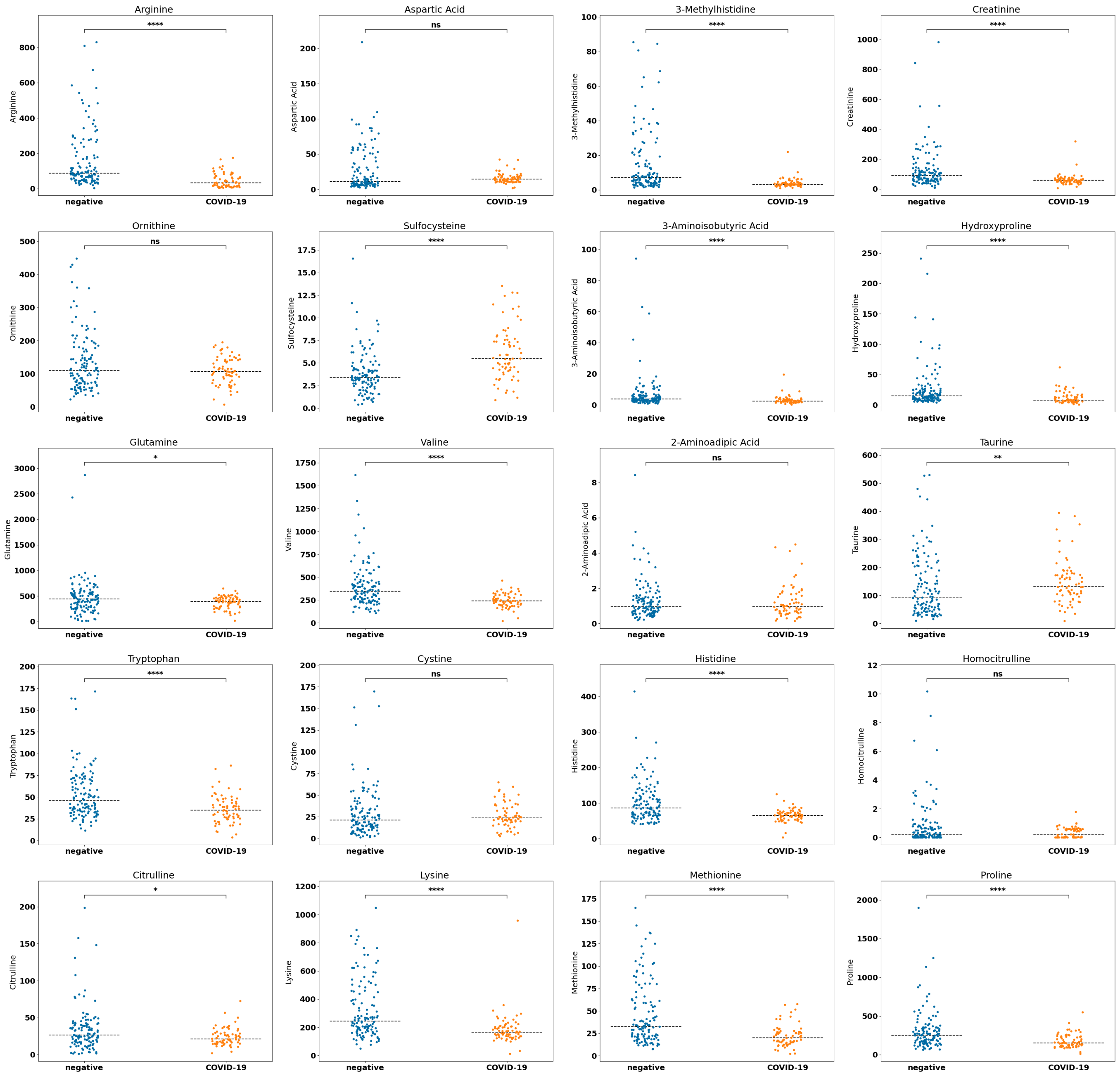
Arginine
****
Aspartic Acid
ns
3-Methylhistidine
****
Creatinine
****
Ornithine
ns
Sulfocysteine
****
3-Aminoisobutyric Acid
****
Hydroxyproline
****
Glutamine
*
Valine
****
2-Aminoadipic Acid
ns
Taurine
**
Tryptophan
****
Cystine
ns
Histidine
****
Homocitrulline
ns
Citrulline
*
Lysine
****
Methionine
****
Proline
****
negative
COVID-19

Supplemental Figure 2. Amino acid concentration by LC/MS-MS standard curve analysis in SARS-CoV-2-positive vs negative specimens for the top 20 differentiating amino acids

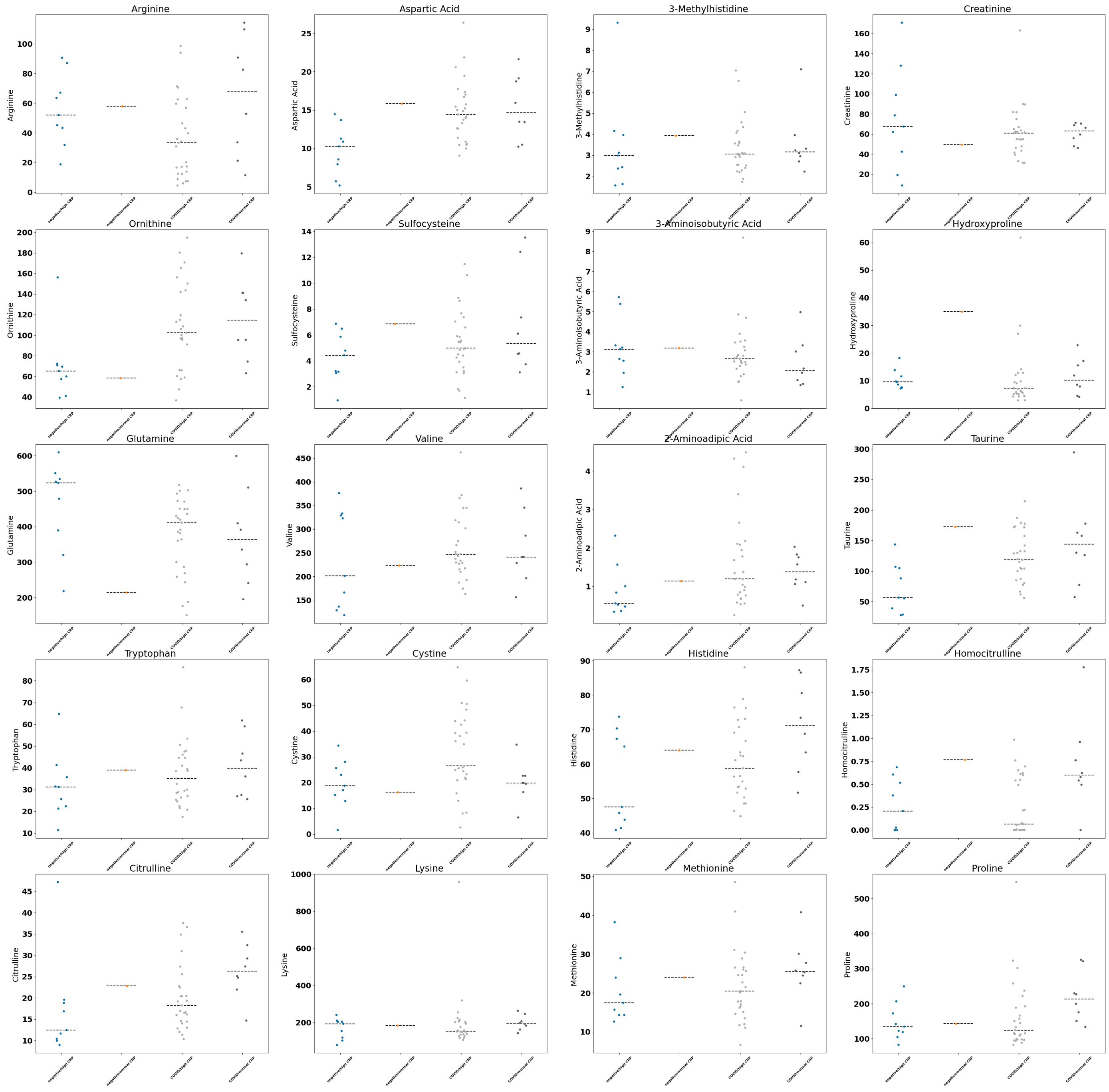
Arginine
Aspartic Acid
3-Methylhistidine
Creatinine
Ornithine
Sulfocysteine
3-Aminoisobutyric Acid
Hydroxyproline
Glutamine
Valine
2-Aminoadipic Acid
Taurine
Tryptophan
Cystine
Histidine
Homocitrulline
Citrulline
Lysine
Methionine
Proline

Supplemental Figure 3. Amino acid concentration by LC/MS-MS standard curve analysis in SARS-CoV-2-positive vs negative specimens for the top 20 differentiating amino acids, stratified by C-reactive protein status. The x-axis categories are listed in the following left to right order: Negative/high CRP, Negative/normal CRP, COVID/high CRP, COVID/normal CRP.

CRP: C-reactive protein
